# Postoperative adjuvant tyrosine kinase inhibitors combined with anti-PD-1 antibodies improves surgical outcomes for hepatocellular carcinoma with high-risk recurrent factors

**DOI:** 10.3389/fimmu.2023.1202039

**Published:** 2023-06-08

**Authors:** Jian Li, Wen-qiang Wang, Rong-hua Zhu, Xing Lv, Jin-lin Wang, Bin-yong Liang, Er-lei Zhang, Zhi-yong Huang

**Affiliations:** Hepatic Surgery Center, Tongji Hospital, Tongji Medical College, Huazhong University of Science and Technology, Wuhan, China

**Keywords:** hepatocellular carcinoma, high-risk recurrent factors, tyrosine kinase inhibitors, anti-PD-1 antibodies, postoperative adjuvant therapy, surgical outcomes

## Abstract

**Background:**

The clinical value of postoperative adjuvant therapy (PAT) for hepatocellular carcinoma (HCC) remains unclear. This study aimed to explore the effect of PAT with tyrosine kinase inhibitors (TKIs) and anti-PD-1 antibodies on the surgical outcomes of HCC patients with high-risk recurrent factors (HRRFs).

**Methods:**

HCC patients who underwent radical hepatectomy at Tongji Hospital between January 2019 and December 2021 were retrospectively enrolled, and those with HRRFs were divided into PAT group and non-PAT group. Recurrence-free survival (RFS) and overall survival (OS) were compared between the two groups after propensity score matching (PSM). Prognostic factors associated with RFS and OS were determined by Cox regression analysis, and subgroup analysis was also conducted.

**Results:**

A total of 250 HCC patients were enrolled, and 47 pairs of patients with HRRFs in the PAT and non-PAT groups were matched through PSM. After PSM, the 1- and 2-year RFS rates in the two groups were 82.1% vs. 40.0% (*P* < 0.001) and 54.2% vs. 25.1% (*P* = 0.012), respectively. The corresponding 1- and 2-year OS rates were 95.4% vs. 69.8% (*P* = 0.001) and 84.3% vs. 55.5% (*P* = 0.014), respectively. Multivariable analyses indicated that PAT was an independent factor related to improving RFS and OS. Subgroup analysis demonstrated that HCC patients with tumor diameter > 5 cm, satellite nodules, or vascular invasion could significantly benefit from PAT in RFS and OS. Common grade 1-3 toxicities, such as pruritus (44.7%), hypertension (42.6%), dermatitis (34.0%), and proteinuria (31.9%) were observed, and no grade 4/5 toxicities or serious adverse events occurred in patients receiving PAT.

**Conclusions:**

PAT with TKIs and anti-PD-1 antibodies could improve surgical outcomes for HCC patients with HRRFs.

## Introduction

Hepatocellular carcinoma (HCC) is one of the most common malignancies and the third leading cause of cancer-related death worldwide (1, 2). At present, hepatectomy remains the preferred treatment for HCC. However, the 5-year recurrence rate after radical hepatectomy in HCC patients is up to 70%, with 5-year survival rate less than 50% (3, 4). Especially for HCC patients with high-risk recurrent factors (HRRFs), such as tumor diameter > 5 cm, multiple tumors, satellite nodules, microvascular invasion (MVI) or portal vein tumor thrombus (PVTT) suffered remarkably higher rate of early recurrence, which directly contributed to worse long-term survival (5–12). Previous studies demonstrated that the 4-month recurrence rate of patients with PVTT was 78.3%, the 1-year recurrence rate of patients with MVI was nearly 50%, while the 6-month recurrence rate of patients with multiple tumors was 60% (5–7). The 5-year survival rates for HCC patients with PVTT, MVI, and multiple tumors were only 32.9%, 33.3%, and 31.9%, respectively (6, 11, 12). Therefore, reducing the early recurrence rate after radical resection is crucial to improve the long-term survival of HCC patients with HRRFs.

Nowadays, there is no uniform and standardized adjuvant treatments to reduce early postoperative recurrence rate in HCC patients (13). Therefore, it is imperative to explore effective strategies to prevent early recurrence of HCC. Previous studies indicated that postoperative adjuvant therapy (PAT) with transcatheter arterial chemoembolization (TACE), hepatic arterial infusion of chemotherapy (HAIC), molecular targeted therapy, adoptive immunotherapy or tumor vaccine could lower the early recurrence rate and further improve the long-term outcomes of HCC patients with HRRFs (14–16). However, the efficacy of the above strategies was still unsatisfactory.

In recent years, molecular targeted therapy combined with immune checkpoint inhibitors (ICIs) have been fruitful for treating advanced HCC (17–19). However, this emerging combination modality has not yet been reported in the settings of adjuvant therapy for HCC patients with HRRFs after radical resection. Several ongoing randomized clinical trials (RCT) exploring the efficacy of the combination therapy in preventing early recurrence of HCC patients with HRRFs undergoing radical resection are still no confirming results (16, 20).

HCC patients with HRRFs after radical resection still had residual small, disseminated foci that could not be removed surgically, which might be responsible for early recurrence and metastasis (21). Tyrosine kinase inhibitors (TKIs) target the vascular endothelial growth factor receptors, with an overall inhibitory effect on tumor angiogenesis (22), thereby potentially clearing the residual micrometastases and preventing tumor recurrence and metastasis after surgery in patients with HRRFs. Anti-programmed death receptor 1 (PD-1) antibodies regulate the immune microenvironment and induce the expansion of T lymphocytes, conducive to the elimination of small metastases in the liver (23). This effect might aid in maximizing the long-term efficacy in patients with HRRFs after radical resection. The combination therapy with TKIs and ICIs represents an attractive therapeutic option to eliminate microscopic tumor foci and disseminated tumor cells. Therefore, we supposed that the combination therapy might reduce the early recurrence rates and further improve the long-term survival in HCC patients with HRRFs.

Herein, this study aimed to investigate whether PAT with TKIs and anti-PD-1 antibodies could improve the surgical outcomes for HCC patients with HRRFs.

## Materials and methods

### Patient selection

This retrospective study included HCC patients who underwent radical resection at the Hepatic Surgery Center, Tongji Hospital, Tongji Medical College, Huazhong University of Science and Technology between January 2019 and December 2021. This study was in line with the requirements of the Helsinki Declaration and was approved by the hospital’s Ethics Committee (TJ-IRB20230127). Due to the retrospective nature of this study, the committee abandoned the requirement of informed consent for all patients.

The inclusion criteria for this study were as follows: (1) age ≥ 18 years; (2) histopathology confirmed HCC; (3) all patients underwent radical hepatectomy according to the Chinese guidelines for diagnosis and treatment of HCC; (4) not receiving any anti-tumor treatment before radical hepatectomy; (5) Child-Pugh grade A or B7. The exclusion criteria for this study were as follows: (1) number of tumors ≥ 4 or extrahepatic metastasis; (2) spontaneous rupture of tumor; (3) PAT with other adjuvant strategies, such as TKIs or anti-PD-1 antibodies monotherapy, TACE; (4) loss to follow-up.

### Data collection and definition

The demographic and clinical features were extracted from the electronic medical record system in our hospital. Vascular invasion was defined as concomitant PVTT or MVI. PVTT mainly included the following types in this study: type I (vp1): tumor thrombus invading the tertiary branch of the portal vein; type II (vp2): tumor thrombus invading the secondary branch of the portal vein; type III (vp3): tumor thrombus invading the primary branch (left or right branch) of the portal vein (24). MVI was defined as nests of cancer cells in the lumen of blood vessels lined by endothelial cells observed microscopically, usually detectable in adjacent liver tissue (25, 26). Satellite nodules were defined as small tumor foci that appear macroscopically or microscopically within the peritumoral liver tissue. The distance between the tumor foci and the primary tumor was < 2 cm. Satellite nodules are considered as intrahepatic micrometastases that occurred on the basis of MVI. They could be classified as MVI, when it was challenging to pathologically distinguish satellite nodules and MVI within the pericancerous liver tissues (26). In this study, HRRFs were defined as including at least one of the following factors: tumor diameter > 5 cm, multiple tumors, satellite nodules, or vascular invasion.

### Surgical resection

All enrolled patients underwent radical hepatectomy, which was defined as: (1) complete resection of tumor nodules found by preoperative imaging or intraoperative exploration, and no cancer cells or residual cancer tissues were detected at the surgical margin of the submitted specimens; (2) no gross tumor thrombus in the main portal vein, hepatic vein, or bile duct, and without intrahepatic or extrahepatic metastasis; (3) no recurrence of HCC within two months after surgery, and for patients with baseline alpha-fetoprotein (AFP) positive, AFP-levels decreased to the normal reference range (26, 27). All operations were performed by the same experienced surgeon with the assistance of other members of the medical team.

All patients received a careful preoperative evaluation. The Eastern Cooperative Oncology Group performance status score assessed the patient’s general condition, and necessary examinations were performed to evaluate the functioning of the vital organs, such as the patients’ heart, lungs and kidneys. The tumor status (location and stage) was judged by color Doppler ultrasound, contrast-enhanced computed tomography (CT), or magnetic resonance imaging (MRI). Indocyanine green retention for 15 min and Child-Pugh grading estimated the liver function reserve. Wide or narrow surgical margin was defined as the shortest distance from the tumor margin to the hepatectomy plane ≥ 1 cm (wide margin) or < 1 cm (narrow margin) (28). Major hepatectomy was defined as the resection of 3 or more liver segments, and minor hepatectomy was defined as the resection of 1 or 2 liver segments (29).

### Postoperative adjuvant therapy

HCC patients with HRRFs would be recommended adjuvant therapies after radical hepatectomy, and the decision ultimately depended on the patient’s own wishes (26). Patients who did not receive adjuvant therapy after hepatectomy were routinely managed according to the guidelines (26, 30). For patients who chose PAT with TKIs and anti-PD-1 antibodies, the TKIs included sorafenib, lenvatinib, donafenib, regorafenib, and apatinib. The anti-PD-1 antibodies included pembrolizumab, sintilimab, camrelizumab, toripalimab, and tislelizumab. TKIs and anti-PD-1 antibodies were administered according to the recommended dosages from four weeks after surgery until HCC recurrence or serious adverse events, or until the patient withdrew automatically. Generally, 3 weeks was taken as one course, and patients in PAT group received at least 3 courses of treatment. Intermittent or reduced dosage was allowed during treatment to reduce drug-related toxicities. Adverse events were classified according to the National Cancer Institute Common Terminology Criteria for Adverse Events version 5.0.

### Postoperative follow-up

All patients were followed up regularly after radical hepatectomy to monitor recurrence, survival status and drug-related toxicities. In the first year of the surgery, blood biochemical (mainly including liver function and AFP), abdominal ultrasound or contrast-enhanced CT/MRI, chest radiograph, or lung CT were performed every two months. In the second year, routine reviews were conducted every 3-4 months and then every half a year. Recurrence was diagnosed according to the typical imaging findings of HCC and/or persistently elevated serum AFP-levels (26). Early recurrence was defined as HCC recurrence within two years after radical resection (31).

Recurrence-free survival (RFS) was defined as the interval between receiving radical hepatectomy and the first diagnosis of recurrence, or the last follow-up. Overall survival (OS) was defined as the period from surgery to the date of death or the last follow-up. The last follow-up date for this study was on August 1, 2022.

### Statistical analysis

Statistical analysis in this study was performed using R software version 4.2.0 (http://www.R-project.org). Continuous variables with normality were presented as mean ± standard deviation (SD), and those without normality were reported as medians and interquartile ranges (IQR). Comparisons between continuous variables were performed using the independent samples *t*-*test* or Mann-Whitney U test, as deemed appropriate. Categorical variables were described as numbers (n) or percentages (%) and compared using the appropriate Chi-square test or Fisher’s exact test. A 1:1 propensity score matching (PSM) was performed to adjust for confounding factors between the two groups. The continuous propensity scores from 0 to 1 were generated by binary logistic regression with selected variables. Nearest-neighbor matching between the PAT and non-PAT groups was done to choose patients for subsequent analyses. The pairs on the propensity-score logit were matched to within a range of 0.2 of SD. Survival curves were created and compared between groups using the Kaplan-Meier method and the log-rank test. Independent prognostic factors for RFS and OS were identified by univariable and multivariable Cox regression analyses. Variables with *P* < 0.05 in the univariable Cox regression were entered into multivariable Cox regression for further analysis. Subgroup survival analyses were conducted using univariable Cox regression, stratified by different clinical variables (gender, age, AFP, number of tumors, tumor diameter, satellite nodules, grade, and vascular invasion), and forest plots were drawn with hazard ratio (HR) and 95% confidence interval (CI). The differences between groups with a two-tailed *P* < 0.05 were considered statistically significant.

## Results

### Baseline characteristics for HCC patients

**Figure 1:**
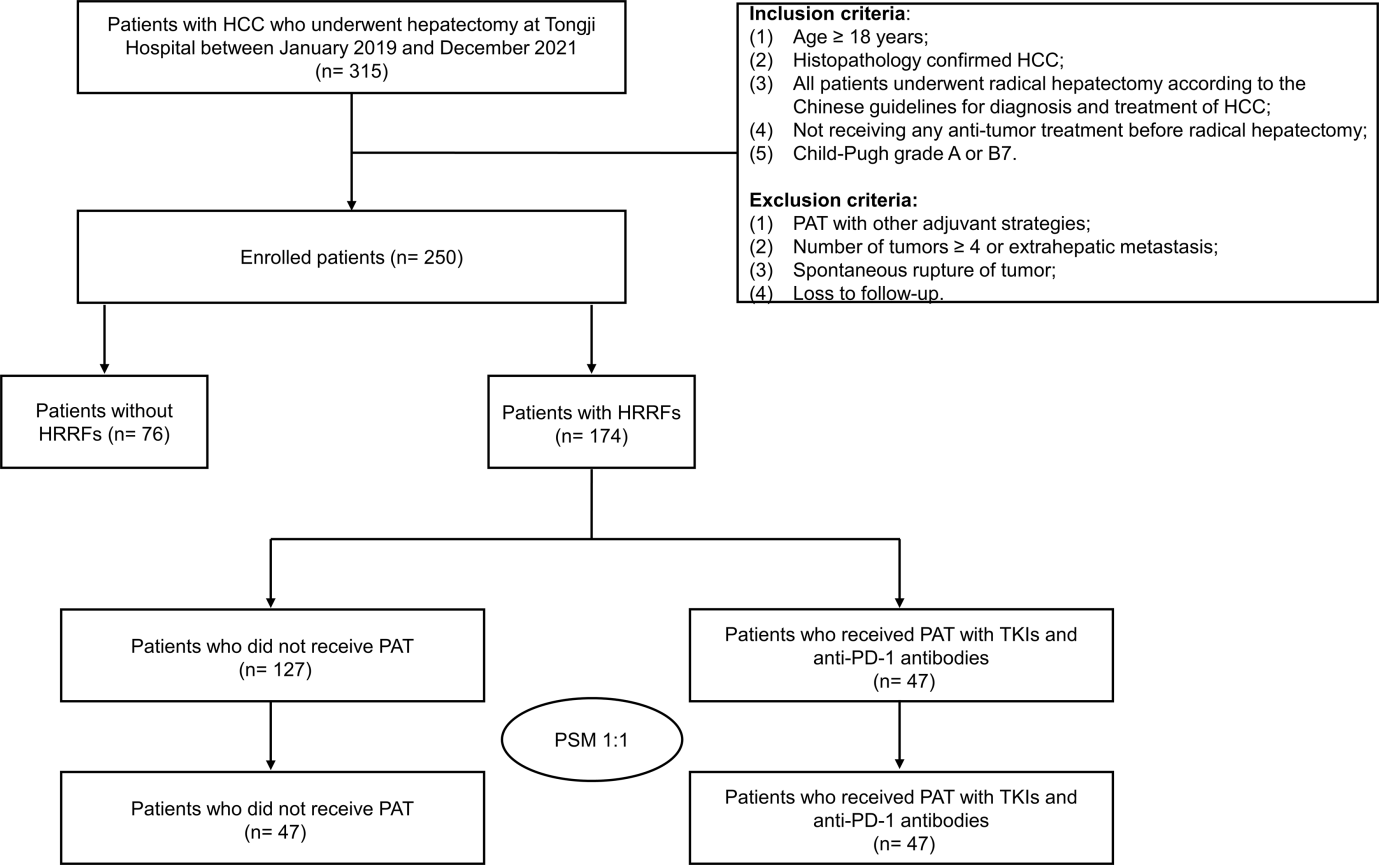
Flow chart of patient selection in this study. HCC, hepatocellular carcinoma; PAT, postoperative adjuvant therapy; TKIs, tyrosine kinase inhibitors; anti-PD-1, anti-programmed death receptor 1; HRRFs, high-risk recurrent factors; PSM, propensity score matching.

A total of 315 HCC patients underwent hepatectomy between January 2019 and December 2021, of whom 65 cases were excluded, including intrahepatic/extrahepatic metastasis (n= 8), spontaneous rupture of HCC (n= 12), previous received hepatectomy, local or systemic treatments (n= 23), PAT with other adjuvant treatments (n= 13), and loss to follow-up (n= 9). Finally, 250 eligible patients were included in this study (**Figure 1**). The clinicopathological characteristics of enrolled patients being depicted in **Table S1**. In these patients, age was 54.3 ± 11.6 years, 89.2% cases were male, 16.8% cases were with multiple tumors, 51.2% cases were with tumor diameter > 5 cm, 25.2% cases were with satellite nodules, and 30.8% cases were with vascular invasion.

### Before and after PSM for HCC patients with HRRFs

As shown in **Table 1**, among the 174 HCC patients with HRRFs before PSM, 47 (27%) were assigned to the PAT group, and 127 (73%) were assigned to the non-PAT group. Significant differences in four variables (AFP, satellite nodules, vascular invasion, and extent of resection) (all *P* < 0.05) were observed upon comparing the baseline characteristics of the two groups. Considering that these confounding factors may interfere with the comparative analysis of survival outcomes between the two groups, PSM was conducted in this study. There were 47 pairs of patients with HRRFs in the PAT and non-PAT groups after PSM, with no significant difference between the two groups (all *P* > 0.05).

**Table 1:** Basal clinicopathological characteristics of 174 HCC patients with HRRFs before and after PSM.

Variable	Before PSM			After PSM		
	Non-PAT group (n= 127)	PAT group (n= 47)	*P*	Non-PAT group (n= 47)	PAT group (n= 47)	*P*
**Gender,**			0.783			1.000
Female	14 (11)	4 (8.5)		5 (10.6)	4 (8.5)	
Male	113 (89)	43 (91.5)		42 (89.4)	43 (91.5)	
**Age,** years			0.387			1.000
< 60	84 (66.1)	35 (74.5)		35 (74.5)	35 (74.5)	
≥ 60	43 (33.9)	12 (25.5)		12 (25.5)	12 (25.5)	
**HBsAg,** IU/mL			0.265			0.830
< 250	57 (44.9)	16 (34)		18 (38.3)	16 (34)	
≥ 250	70 (55.1)	31 (66)		29 (61.7)	31 (66)	
**HBV-DNA,** copies/mL			0.940			.000
< 2000	92 (72.4)	35 (74.5)		35 (74.5)	35 (74.5)	
≥ 2000	35 (27.6)	12 (25.5)		12 (25.5)	12 (25.5)	
**PLT,** x 10^9^/L			0.255			0.714
≤ 100	18 (14)	3 (6.4)		5 (10.6)	3 (6.4)	
> 100	109 (86)	44 (93.6)		42 (89.4)	44 (93.6)	
**PT,** seconds			0.606			0.450
≤ 14.5	99 (78)	39 (83)		35 (74.5)	39 (83)	
> 14.5	28 (22)	8 (17)		12 (25.5)	8 (17)	
**ALT,** U/L			0.390			0.825
≤ 40	94 (74)	31 (66)		33 (70.2)	31 (66)	
> 40	33 (26)	16 (34)		14 (29.8)	16 (34)	
**AST,** U/L			1.000			0.499
≤ 40	95 (74.8)	35 (74.5)		31 (66)	35 (74.5)	
> 40	32 (25.2)	12 (25.5)		16 (34)	12 (25.5)	
**ALB,** g/L			0.208			0.738
≤ 35	8 (6)	6 (12.8)		4 (8.5)	6 (12.8)	
> 35	119 (94)	41 (87.2)		43 (91.5)	41 (87.2)	
**TBIL,** µmol/L			0.879			1.000
≤ 20	105 (83)	40 (85.1)		39 (83)	40 (85.1)	
> 20	22 (17)	7 (14.9)		8 (17)	7 (14.9)	
**AFP,** ng/mL			**0.001**			1.000
< 400	90 (70.9)	20 (42.6)		20 (42.6)	20 (42.6)	
≥ 400	37 (29.1)	27 (57.4)		27 (57.4)	27 (57.4)	
**Number of tumors**			0.951			1.000
Single	97 (76.4)	35 (74.5)		34 (72.3)	35 (74.5)	
Multiple	30 (23.6)	12 (25.5)		13 (27.7)	12 (25.5)	
**Tumor diameter,** cm			0.977			1.000
≤ 5	33 (26)	13 (27.7)		13 (27.7)	13 (27.7)	
> 5	94 (74)	34 (72.3)		34 (72.3)	34 (72.3)	
**Satellite nodules,**			**0.021**			1.000
No	88 (69.3)	23 (48.9)		23 (48.9)	23 (48.9)	
Yes	39 (30.7)	24 (51.1)		24 (51.1)	24 (51.1)	
**Edmondson-Steiner grade,**			0.125			1.000
I-II	56 (44.1)	14 (29.8)		13 (27.7)	14 (29.8)	
III-IV	71 (55.9)	33 (70.2)		34 (72.3)	33 (70.2)	
**Vascular invasion,**			**< 0.001**			1.000
No	83 (65.4)	14 (29.8)		15 (31.9)	14 (29.8)	
Yes	44 (34.6)	33 (70.2)		32 (68.1)	33 (70.2)	
**Blood loss,** mL			0.571			1.000
< 400	104 (82)	36 (76.6)		37 (78.7)	36 (76.6)	
≥ 400	23 (18)	11 (23.4)		10 (21.3)	11 (23.4)	
**Transfusion,**			1.000			1.000
No	119 (94)	44 (93.6)		45 (95.7)	44 (93.6)	
Yes	8 (6)	3 (6.4)		2 (4.3)	3 (6.4)	
**Margin,**			0.186			0.620
Narrow	39 (30.7)	9 (19.1)		12 (25.5)	9 (19.1)	
Wide	88 (69.3)	38 (80.9)		35 (74.5)	38 (80.9)	
**Extent of resection,**			**0.042**			0.283
Minor	95 (74.8)	27 (57.4)		33 (70.2)	27 (57.4)	
Major	32 (25.2)	20 (42.6)		14 (29.8)	20 (42.6)	

Bold values indicate statistical significance (*P* < 0.05).HCC, hepatocellular carcinoma; HRRFs, high-risk recurrent factors; PSM, propensity score matching; PAT, postoperative adjuvant therapy; HBsAg, hepatitis B surface antigen; HBV-DNA, hepatitis B virus-deoxyribonucleic acid; PLT, platelet; PT, prothrombin time; ALT, alanine aminotransferase; AST, aspartate aminotransaminase; ALB, serum albumin; TBIL, total serum bilirubin; AFP, alpha-fetoprotein.

### Survival analysis

**Figure 2:**
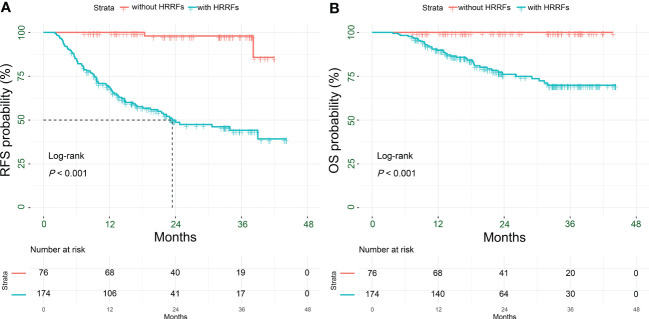
Kaplan-Meier analysis for survival outcomes in HCC patients with or without HRRFs after radical resection. RFS **(A)** and OS **(B)** for patients. HCC, hepatocellular carcinoma; HRRFs, high-risk recurrent factors; RFS, recurrence-free survival; OS, overall survival.

The median follow-up for the 250 HCC patients was 22.4 (IQR: 14.3-34.1) months, including 84 (33.6%) recurrences and 39 (15.6%) deaths; and the 90-day mortality was 0 (**Table S1**). As shown in **Figures 2A, B**, the RFS and OS of patients without HRRFs were significantly longer than those with HRRFs (all *P* < 0.001). Moreover, before PSM, there was no significant difference in RFS and OS between PAT and non-PAT groups of HCC patients with HRRFs. However, the OS of the PAT group of patients tended to be longer than those of the non-PAT group (all *P* > 0.05, **Figure S1**). This observation might be attributed to the imbalance of baseline characteristics of the two groups of patients. In contrast, the PAT group patients had higher tumor malignancy (AFP ≥ 400 ng/mL, satellite nodules, and vascular invasion) than those in the non-PAT group.

**Figure 3:**
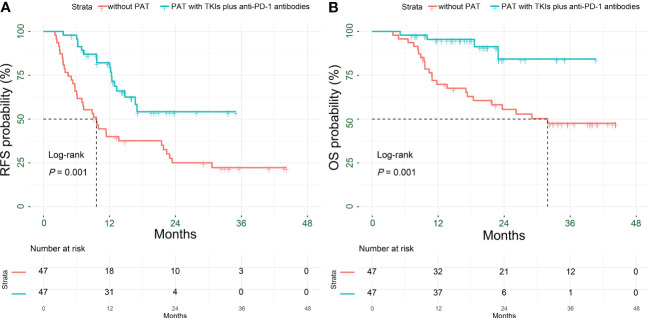
Kaplan-Meier analysis for survival outcomes in HCC patients with HRRFs who underwent radical resection after PSM. RFS **(A)** and OS **(B)** for patients. HCC, hepatocellular carcinoma; HRRFs, high-risk recurrent factors; RFS, recurrence-free survival; OS, overall survival; PSM, propensity score matching; PAT, postoperative adjuvant therapy; TKIs, tyrosine kinase inhibitors; anti-PD-1, anti-programmed death receptor 1.

After PSM, there were 17 patients (36.2%), 35 patients (74.5%) recurrences (*P* < 0.001), and 4 patients (8.5%), 23 patients (48.9%) deaths (*P* < 0.001) in the in PAT and non-PAT groups, respectively. Patients in the PAT group had significantly longer RFS and OS than those in the non-PAT group (all *P* < 0.05). The 1- and 2-year RFS rates of the patients in the two groups were 82.1% vs. 40.0% (*P* < 0.001) and 54.2% vs. 25.1% (*P* = 0.012), respectively; and the corresponding 1- and 2-year OS rates were 95.4% vs. 69.8% (*P* = 0.001) and 84.3% vs. 55.5% (*P* = 0.014), respectively (**Figure 3** **,** **Table 2**).

**Table 2:** Survival features of HCC patients with HRRFs in various subgroups after PSM.

Patients in various subgroups	Non-PAT group	PAT group	*P*
**All patients,** (n= 94)	47	47	–
1 year-RFS (%)	40.0	82.1	**< 0.001**
2 year-RFS (%)	25.1	54.2	**0.012**
Recurrence rates (%)	74.5 (35/47)	36.2 (17/47)	**< 0.001**
1 year-OS (%)	69.8	95.4	**0.001**
2 year-OS (%)	55.5	84.3	**0.014**
3 year-OS (%)	47.6	84.3	**0.002**
Death rates (%)	48.9 (23/47)	8.5 (4/47)	**< 0.001**
Follow-up time (median, months)	22.4	18.1	–
**Patients with multiple tumors,** (n= 25)	13	12	–
1 year-RFS (%)	28.8	75.0	**0.015**
Recurrence rates (%)	76.9 (10/13)	33.3 (4/12)	0.073
1 year-OS (%)	59.2	100	**0.006**
2 year-OS (%)	32.9	66.7	0.227
Death rates (%)	53.9 (7/13)	8.3 (1/12)	**0.030**
Follow-up time (median, months)	14.8	16.4	–
**Patients with tumor diameter > 5cm,** (n= 68)	34	34	–
1 year-RFS (%)	38.2	78.7	**< 0.001**
2 year-RFS (%)	20.9	46.5	**0.048**
Recurrence rates (%)	79.4 (27/34)	44.1 (15/34)	**0.006**
1 year-OS (%)	61.8	96.7	**< 0.001**
2 year-OS (%)	48.7	82.2	**0.018**
Death rates (%)	55.9 (19/34)	8.8 (3/34)	**< 0.001**
Follow-up time (median, months)	17.7	19.3	–
**Patients with satellite nodules,** (n= 48)	24	24	–
1 year-RFS (%)	25.0	82.6	**< 0.001**
2 year-RFS (%)	15.0	32.8	0.220
Recurrence rates (%)	83.3 (20/24)	41.7 (10/24)	**0.007**
1 year-OS (%)	58.3	100	**< 0.001**
2 year-OS (%)	35.9	71.1	0.093
Death rates (%)	75.0 (18/24)	8.3 (2/24)	**< 0.001**
Follow-up time (median, months)	15.3	15.5	–
**Patients with vascular invasion,** (n= 65)	32	33	–
1 year-RFS (%)	40.0	80.2	**< 0.001**
2 year-RFS (%)	32.0	62.2	**0.028**
Recurrence rates (%)	68.8 (22/32)	30.3 (10/33)	**0.004**
1 year-OS (%)	71.4	93.5	**0.022**
2 year-OS (%)	56.5	93.5	**< 0.001**
Death rates (%)	40.6 (13/32)	6.1 (2/33)	**0.003**
Follow-up time (median, months)	22.1	19.6	–

Bold values indicate statistical significance (*P* < 0.05).HCC, hepatocellular carcinoma; HRRFs, high-risk recurrent factors; PSM, propensity score matching; PAT, postoperative adjuvant therapy; RFS, recurrence-free survival; OS, overall survival.

### Subgroup survival analysis

**Figure 4:**
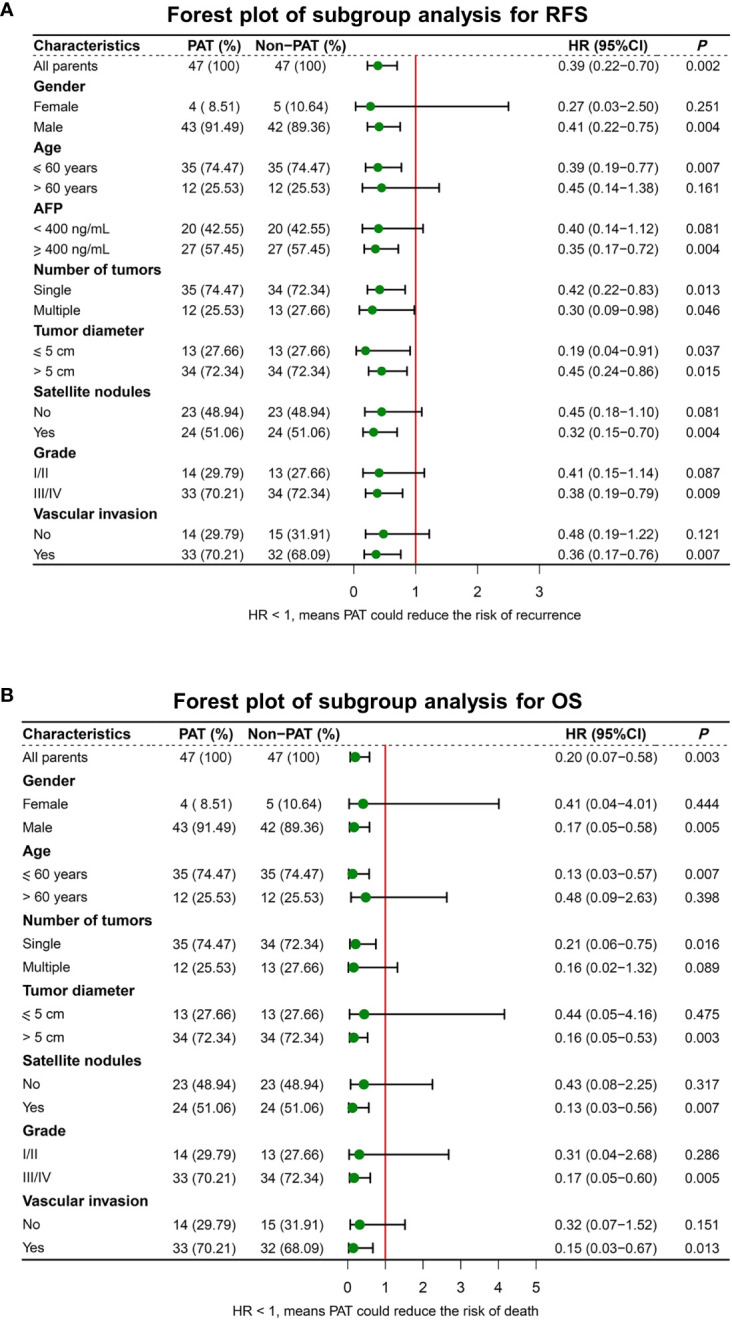
Forest plot for subgroup survival analysis for RFS **(A)** and OS **(B)**. RFS, recurrence-free survival; OS, overall survival; PAT, postoperative adjuvant therapy; AFP, alphafetoprotein.

To further explore the potential value of PAT for improving RFS and OS, subgroup analyses were performed. The results indicated that PAT could significantly improve RFS in HCC patients with multiple tumors (HR: 0.30, 95% CI: 0.09-0.98, *P* = 0.046), tumor diameter > 5 cm (HR: 0.45, 95% CI: 0.24-0.86, *P* = 0.015), satellite nodules (HR: 0.32, 95% CI: 0.15-0.70, *P* = 0.004) or vascular invasion (HR: 0.36, 95% CI: 0.17-0.76, *P* = 0.007) (**Figure 4A**).

In addition, PAT significantly improved OS in HCC patients with tumor diameter > 5 cm (HR: 0.16, 95% CI: 0.05-0.53, *P* = 0.003), satellite nodules (HR: 0.13, 95% CI: 0.03-0.56, *P* = 0.007) or vascular invasion (HR: 0.15, 95% CI: 0.03-0.67, *P* = 0.013) (**Figure 4B**).

**Figure 5:**
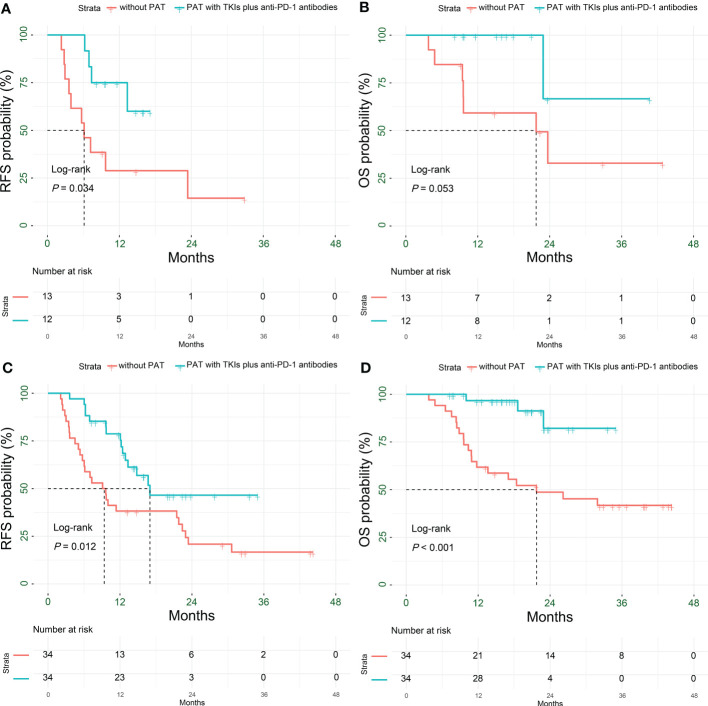
Kaplan-Meier subgroup analysis for survival outcomes in HCC patients with HRRFs who underwent radical resection after PSM. RFS **(A)** and OS **(B)** for patients with multiple tumors; RFS **(C)** and OS **(D)** for patients with tumor diameter > 5 cm. HCC, hepatocellular carcinoma; HRRFs, high-risk recurrent factors; RFS, recurrence-free survival; OS, overall survival; PSM, propensity score matching; PAT, postoperative adjuvant therapy; TKIs, tyrosine kinase inhibitors; anti-PD-1, anti-programmed death receptor 1.

PAT significantly prolonged the RFS (*P* = 0.034), and relatively extended the OS (*P* = 0.053) for HCC patients with multiple tumors. The 1-year RFS rate of patients in the PAT and non-PAT groups was 75% and 28.8%, respectively (*P* = 0.015); and the 1- and 2-year OS rates were 100% vs. 59.2% (*P* = 0.006) and 66.7% vs. 32.9% (*P* = 0.227), respectively (**Figures 5A, B**, **Table 2**).

PAT significantly improved the RFS and OS for HCC patients with tumor diameter > 5 cm (*P*= 0.012 and *P* < 0.001, respectively). The 1- and 2-year RFS rates of patients in the two groups were 78.7% vs. 38.2% (*P* < 0.001) and 46.5% vs. 20.9% (*P* = 0.048), respectively; while the 1- and 2-year OS rates were 96.7% vs. 61.8% (*P* < 0.001) and 82.2% vs. 48.7% (*P* = 0.018), respectively (**Figures 5C, D**, **Table 2**).

**Figure 6:**
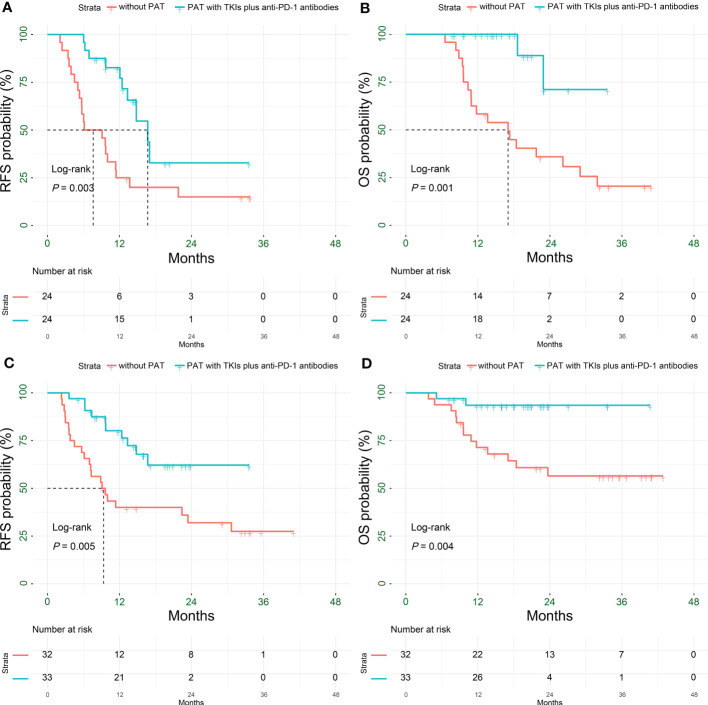
Kaplan-Meier subgroup analysis for survival outcomes in HCC patients with HRRFs who underwent radical resection after PSM. RFS **(A)** and OS **(B)** for patients with satellite nodules; RFS **(C)** and OS **(D)** for patients with vascular invasion. HCC, hepatocellular carcinoma; HRRFs, high-risk recurrent factors; RFS, recurrence-free survival; OS, overall survival; PSM, propensity score matching; PAT, postoperative adjuvant therapy; TKIs, tyrosine kinase inhibitors; anti-PD-1, anti-programmed death receptor 1.

The RFS and OS of HCC patients with satellite nodules in the PAT group were significantly longer than those in the non-PAT group (*P* = 0.003 and *P* = 0.001, respectively). The 1- and 2-year RFS rates of patients in the two groups were 82.6% vs. 25% (*P* < 0.001) and 32.8% vs. 15% (*P* = 0.220), respectively. The 1- and 2-year OS were 100% vs. 58.3% (*P* < 0.001) and 71.1% vs. 35.9% (*P* = 0.093), respectively (**Figures 6A, B**, **Table 2**).

The RFS and OS of patients in the PAT group were significantly longer than those of HCC patients with vascular invasion in the non-PAT group (*P* = 0.005 and *P* = 0.004, respectively). The 1- and 2-year RFS rates were 80.2% vs. 40% (*P* < 0.001) and 62.2% vs. 32% (*P* = 0.028), respectively. The 1- and 2-year OS rates were 93.5% vs. 71.4% (*P* = 0.022) and 93.5% vs. 56.5% (*P* < 0.001), respectively (**Figures 6C, D**, **Table 2**).

### Independent prognostic factors related to RFS and OS

Multivariable Cox regression analysis indicated two variables to closely associated with RFS in HCC patients with HRRFs: AFP ≥ 400 ng/mL (HR: 2.28, 95% CI: 1.27-4.11, *P* = 0.006) and PAT (HR: 0.33, 95% CI: 0.18-0.60, *P* < 0.001) (**Table S2**). Moreover, three factors were strongly related to OS in HCC patients with HRRFs: AFP ≥ 400 ng/mL (HR: 2.91, 95% CI: 1.19-7.11, *P =* 0.019), satellite nodules (HR: 2.76, 95% CI: 1.05-7.22, *P* = 0.039), and PAT (HR: 0.18, 95% CI: 0.06-0.54, *P* = 0.002) (**Table S3**). The above results significantly established PAT as a favorable factor in improving the RFS and OS of HCC patients with HRRFs.

### Safety

The occurrence of adverse events in 47 patients receiving PAT was summarized in **Table 3**. No grade 4/5 toxicities or serious adverse events were observed, and common grade 1-3 toxicities included pruritus (44.7%), hypertension (42.6%), dermatitis (34.0%), and proteinuria (31.9%). Grade 3 toxicities included dermatitis (6.4%), diarrhea (6.4%), pruritus (2.1%), hypertension (2.1%), hand-foot skin reaction (2.1%), proteinuria (2.1%), and thrombocytopenia (2.1%).

**Table 3:** Adverse events of the PAT with TKIs and anti-PD-1 antibodies and their CTCAE grade.

Adverse events	PAT with TKIs and anti-PD-1 antibodies (n= 47)			
	All (%)	Grade 1 (%)	Grade 2 (%)	Grade 3 (%)
Clinical symptoms				
Pruritus	44.7 (21/47)	31.9 (15/47)	10.6 (5/47)	2.1 (1/47)
Hypertension	42.6 (20/47)	31.9 (15/47)	8.5 (4/47)	2.1 (1/47)
Dermatitis	34.0 (16/47)	19.1 (9/47)	8.5 (4/47)	6.4 (3/47)
Fatigue	25.5 (12/47)	19.1 (9/47)	6.4 (3/47)	0
Anorexia	21.3 (10/47)	12.8 (6/47)	8.5 (4/47)	0
Diarrhea	19.1 (9/47)	8.5 (4/47)	4.2 (2/47)	6.4 (3/47)
HRSR	19.1 (9/47)	6.4 (3/47)	10.6 (5/47)	2.1 (1/47)
Stomatitis	10.6 (5/47)	6.4 (3/47)	4.2 (2/47)	0
Dysphonia	6.4 (3/47)	4.2 (2/47)	2.1 (1/47)	0
Pneumonitis	6.4 (3/47)	6.4 (3/47)	0	0
Laboratory test				
Proteinuria	31.9 (15/47)	21.3 (10/47)	8.5 (4/47)	2.1 (1/47)
Neutropenia	27.7 (13/47)	25.6 (12/47)	2.1 (1/47)	0
Thrombocytopenia	25.6 (12/47)	21.3 (10/47)	2.1 (1/47)	2.1 (1/47)
Anemia	25.6 (12/47)	23.4 (11/47)	2.1 (1/47)	0
Hypothyroidism	21.3 (10/47)	19.1 (9/47)	2.1 (1/47)	0

PAT, postoperative adjuvant therapy; TKIs, tyrosine kinase inhibitors; anti-PD-1, anti-programmed cell death protein 1; CTCAE, common terminology criteria for adverse events; HRSR, hand-foot skin reaction.

## Discussion

Nowadays, the administration of adjuvant therapy to HCC patients after radical resection is still highly debatable. Western guidelines suggested that adjuvant therapy should not be routinely recommended for HCC patients undergoing radical resection due to a lack of solid evidence demonstrating adjuvant therapy improving survival outcomes (32, 33). A large-sample RCT (STORM, phase III trial) result indicated that PAT with sorafenib failed to effectively improve the RFS and OS in patients with HCC (34). On the contrary, several findings revealed that PAT with sorafenib extended RFS and OS for patients with high recurrence risk (35–38). The ending of the STORM trial with negative results might be attributed to the inclusion of early-stage HCC patients with low recurrence risk in the study, making it difficult to observe differences in the survival curves of patients between the adjuvant sorafenib group and the control group.

This study found that HCC patients with HRRFs were more likely to experience early recurrence and suffered worse survival prognosis than those without HRRFs. Previous studies also confirmed that higher rates of early recurrence and metastasis after curative resection in HCC patients with HRRFs, severely affecting their long-term survival (5–7, 39). Accordingly, Asian Pacific scholars advocated that for HCC patients with HRRFs, adjuvant therapeutic measures should be taken after curative resection to prevent early recurrence and metastasis (26, 40).

As far as we know, the results of this study revealed for the first time that PAT using TKIs in combination with anti-PD-1 antibodies showed a strong association with reduced early recurrence rate and prolonged OS in HCC patients with HRRFs. Compared with the non-PAT group, the early recurrence rate of patients in the PAT group could be reduced by 38.3%, and the 3-year OS rate could be increased by 36.7%. To avoid the interference of confounding factors (such as AFP, satellite nodules, vascular invasion, etc.) on RFS and OS, this study balanced the baseline characteristics of the two groups through 1:1 PSM. Therefore, the markedly lower risk of early recurrence and the significantly prolonged OS should be attributed to PAT. In addition, multivariable results showed that PAT was an independent prognostic factor for improving RFS and OS. This finding of the present study sufficiently demonstrates that PAT has great potential to be an effective modality for HCC patients with HRRFs to prevent early recurrence and prolong the long-term survival.

An RCT by Wang et al. indicated that adjuvant TACE only reduced the early recurrence rate by 15.8% and improved the 3-year OS rate by 7.8% for patients with HRRFs (15). Another multicenter RCT result showed that PAT with HAIC could only lower the recurrence rate by 15.6% and increase the 3-year OS rate by 5.5% in patients with MVI (41). Zhang et al. reported that adjuvant sorafenib could reduce the early recurrence rate by 17% and improve the 3-year OS rate by 15% for patients with MVI (38). A recent study illustrated that adjuvant lenvatinib could improve the early RFS rates of patients with MVI by 17.1%-27.3% (42). Chen et al. demonstrated that adjuvant anti-PD-1 antibodies ameliorated the early RFS rates by 22.8%-24.4% in patients with HRRFs (43). However, the results of this study indicated that PAT using TKIs combined with anti-PD-1 antibodies could improve the early RFS rates by 29.1%-42.1% in patients with HRRFs. Compared with adjuvant TACE, HAIC, or TKIs and anti-PD-1 antibodies monotherapy, adjuvant TKIs combined with anti-PD-1 antibodies have shown an overwhelming advantage in improving the survival prognosis of patients with HRRFs. This may be attributed to the synergistic anti-tumor effects of the combined regimens, which is more beneficial for clearing the residual micrometastatic lesions in the remaining liver, thus achieving satisfactory effects of preventing early recurrence and prolonging long-term survival (43–46). Notably, the present study showed that the combination therapy would not increase the probability of treatment-related adverse events compared with monotherapy (34, 42, 43).

Early recurrence (≤ 2 years) is the most common type of HCC recurrence, mainly related to the biologic features of tumor (such as tumor diameter > 5 cm, multiple tumors, satellite nodules, vascular invasion, or poor differentiation, etc.), while late recurrence (> 2 years) is generally considered to be associated with the underlying liver disease background (31). PAT with TKIs and anti-PD-1 antibodies could effectively control early recurrence caused by the dissemination of intrahepatic micrometastatic lesions, but its curative effect on late recurrence might be limited owing to the inability to alter the underlying liver diseases. The median follow-up was relatively short (22.4 months), so this study mainly observed the influencing factors of early recurrence. In the overall cohort, multivariable results showed that tumor diameter > 5 cm, multiple tumors, satellite nodules, and vascular invasion were high-risk factors associated with early recurrence, which was consistent with previous studies (5, 7, 9) (**Table S4**). Excitingly, subgroup analysis suggested that PAT could significantly improve RFS and OS for patients with tumor diameter > 5 cm, satellite nodules, or vascular invasion. PAT failed to result in a significant improvement in OS for patients with multiple tumors, which might be related to the insufficient sample size of subgroups.

This study had several limitations. First, although PSM and multivariable analysis were adopted, the influence of selection bias on the findings could not be completely excluded due to the inherent deficiencies of single center, retrospective design. Therefore, the conclusion of this study still needs to be further validated by several ongoing RCT results. Second, the sample size of this study was limited, and the duration of follow-up was relatively short, thus the 5-year survival results were unavailable. Therefore, future studies still need to expand the sample size and extend the follow-up time. Third, this study was limited to clinical observation, and the mechanism of postoperative adjuvant combination therapy to prevent early recurrence in HCC patients with HRRFs deserves thorough exploration.

## Conclusions

In conclusion, the present study demonstrated that adjuvant TKIs combined with anti-PD-1 antibodies after radical resection closely associated with improved surgical outcomes for HCC patients with HRRFs. Moreover, the adverse events of adjuvant therapy were controllable, providing more confidence in using this line of therapy. However, multicenter studies with large-sample sizes still need to be carried out to validate this study’s results further.

## Data availability statement

The raw data supporting the conclusions of this article will be made available by the authors, without undue reservation.

## Ethics statement

This study was approved by the Tongji Hospital Ethics Committee (TJ-IRB20230127). Due to the retrospective nature of this study, the committee waived the requirement of informed consent for all patients.

## Author contributions

Conception and design, Z-YH, E-LZ, and JL. Administrative support, Z-YH, E-LZ, and B-YL. Provision of study materials or patients, Z-YH, E-LZ, and B-YL. Collection and assembly of data, JL, W-QW, XL, J-LW, and R-HZ. Data analysis and interpretation, JL, E-LZ, and Z-YH. All authors contributed to the article and approved the submitted version.
