# Supplementary material for: Postoperative adjuvant tyrosine kinase inhibitors combined with anti-PD-1 antibodies improves surgical outcomes for hepatocellular carcinoma with high-risk recurrent factors
Source: Front Immunol. 2023 Jun 8;14:1202039. doi: 10.3389/fimmu.2023.1202039 (PMC10285103; doi:10.3389/fimmu.2023.1202039)
Supplement: Supplementary file 1 [file DataSheet_1.zip › Supplementary Figure 1.DOCX]

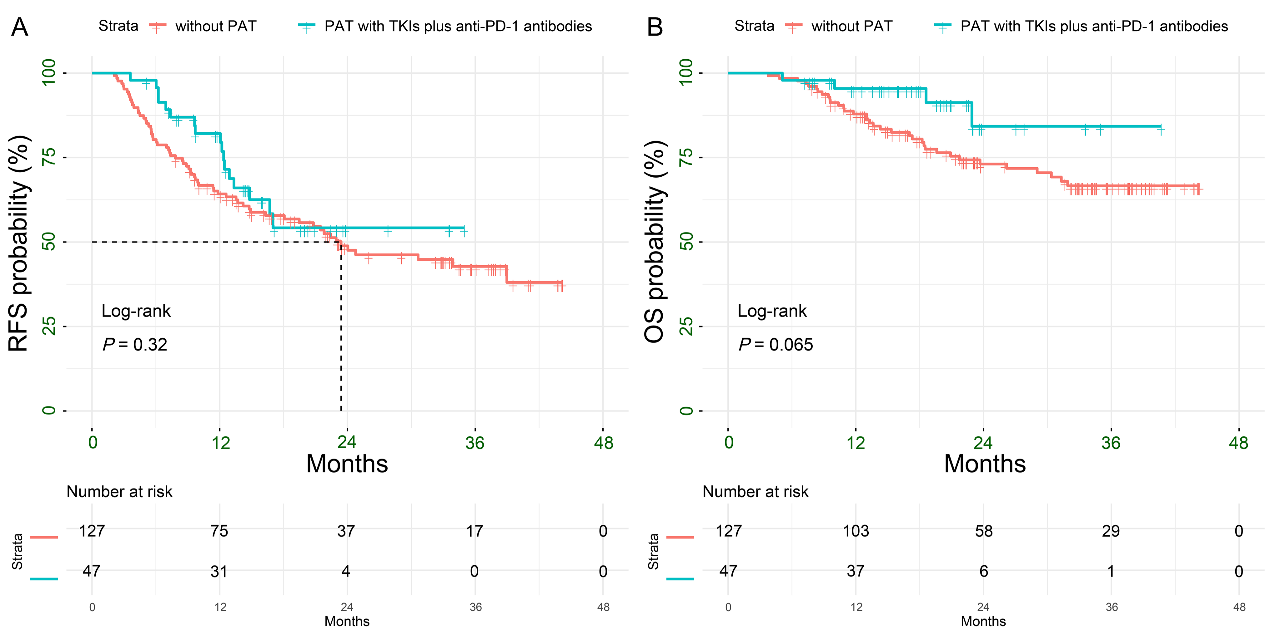


**FIGURE S1** Kaplan-Meier analysis for survival outcomes in HCC patients with HRRFs who underwent radical resection before PSM. RFS **(A)** and OS **(B)** for patients. HCC, hepatocellular carcinoma; HRRFs, high-risk recurrent factors; RFS, recurrence-free survival; OS, overall survival; PSM, propensity score matching; PAT, postoperative adjuvant therapy; TKIs, tyrosine kinase inhibitors; anti-PD-1, anti-programmed death receptor 1.
