# Supplementary material for: Postoperative adjuvant tyrosine kinase inhibitors combined with anti-PD-1 antibodies improves surgical outcomes for hepatocellular carcinoma with high-risk recurrent factors
Source: Front Immunol. 2023 Jun 8;14:1202039. doi: 10.3389/fimmu.2023.1202039 (PMC10285103; doi:10.3389/fimmu.2023.1202039)
Supplement: Supplementary file 1 [file DataSheet_1.zip › Supplementary Table 1.DOCX]

**TABLE S1** Baseline characteristics for 250 HCC patients.

| **Variable** |  |
| --- | --- |
| **Gender**, N (%) |  |
| Female | 27 (10.8) |
| Male | 223 (89.2) |
| **Age** ^a^, years | 54.3 ± 11.6 |
| **HBsAg** (IU/mL) , N (%) |  |
| < 250 | 109 (43.6) |
| ≥ 250 | 141 (56.4) |
| **HBV-DNA** (copies/mL) , N (%) |  |
| < 2000 | 186 (74.4) |
| ≥ 2000 | 64 (25.6) |
| **PLT** ^b^, x 10^9^/L | 163.0 (115.8-218.5) |
| **PT** ^b^, seconds | 13.7 (13.2-14.3) |
| **ALT** ^b^, U/L | 25.0 (18.0-42.0) |
| **AST** ^b^, U/L | 29.0 (21.0-39.0) |
| **ALB** ^b^, g/L | 40.3 (38.0-43.2) |
| **TBIL** ^b^, µmol/L | 12.7 (9.3-16.8) |
| **AFP** (ng/mL) , N (%) |  |
| < 400 | 177 (70.8) |
| ≥ 400 | 73 (29.2) |
| **Number of tumors**, N (%) |  |
| Single | 208 (83.2) |
| Multiple | 42 (16.8) |
| **Tumor diameter** ^b^, cm | 5.1 (3.5-7.8) |
| **Tumor diameter** (cm) , N (%) |  |
| ≤ 5 | 122 (48.8) |
| > 5 | 128 (51.2) |
| **Satellite** **nodules**, N (%) |  |
| No | 187 (74.8) |
| Yes | 63 (25.2) |
| **Edmondson-Steiner grade**, N (%) |  |
| I-II | 123 (49.2) |
| III-IV | 127 (50.8) |
| **Vascular invasion**, N (%) |  |
| No | 173 (69.2) |
| Yes | 77 (30.8) |
| **Blood loss** (mL), N (%) |  |
| < 400 | 215 (86.0) |
| ≥ 400 | 35 (14.0) |
| **Transfusion**, N (%) |  |
| No | 238 (95.2) |
| Yes | 12 (4.80) |
| **Margin**, N (%) |  |
| Narrow | 71 (28.4) |
| Wide | 179 (71.6) |
| **Extent of resection**, N (%) |  |
| Minor | 196 (78.4) |
| Major | 54 (21.6) |
| **PAT**, N (%) |  |
| No | 203 (81.2) |
| Yes | 47 (18.8) |
| **Recurrence**, N (%) |  |
| No | 166 (66.4) |
| Yes | 84 (33.6) |
| **Dead**, N (%) |  |
| No | 211 (84.4) |
| Yes | 39 (15.6) |
| **The 90-day mortality**, (%) | 0 |
| **Follow-up time** ^b^, months | 22.4 (14.3-34.1) |

^a^ Continuous variables were presented as mean ± standard deviation. ^b^ Continuous variables were presented as medians and interquartile ranges. HCC, hepatocellular carcinoma; HBsAg, hepatitis B surface antigen; HBV-DNA, hepatitis B virus-deoxyribonucleic acid; PLT, platelet; PT, prothrombin time; ALT, alanine aminotransferase; AST, aspartate aminotransaminase; ALB, serum albumin; TBIL, total serum bilirubin; AFP, alpha-fetoprotein; PAT, postoperative adjuvant therapy.
