# Supplementary material for: Postoperative adjuvant tyrosine kinase inhibitors combined with anti-PD-1 antibodies improves surgical outcomes for hepatocellular carcinoma with high-risk recurrent factors
Source: Front Immunol. 2023 Jun 8;14:1202039. doi: 10.3389/fimmu.2023.1202039 (PMC10285103; doi:10.3389/fimmu.2023.1202039)
Supplement: Supplementary file 1 [file DataSheet_1.zip › Supplementary Table 2.DOCX]

**TABLE S2** Univariable and multivariable Cox regression analyses for risk factors associated with RFS in HCC patients with HRRFs after PSM.

| **Variable** | **Univariable Analysis** | | **Multivariable Analysis** | |
| --- | --- | --- | --- | --- |
|  | **HR (95% CI)** | ***P*** | **HR (95% CI)** | ***P*** |
| **Gender**, |  |  |  |  |
| Male vs. female | 0.86 (0.37-2.01) | 0.725 |  |  |
| **Age**, years |  |  |  |  |
| ≥ 60 vs. < 60 | 0.93 (0.50-1.75) | 0.828 |  |  |
| **HBsAg**, IU/mL |  |  |  |  |
| ≥ 250 vs. < 250 | 0.86 (0.49-1.49) | 0.587 |  |  |
| **HBV-DNA**, copies/mL |  |  |  |  |
| ≥ 2000 vs. < 2000 | 0.89 (0.46-1.69) | 0.714 |  |  |
| **PLT**, x 10^9^/L |  |  |  |  |
| > 100 vs. ≤ 100 | 0.79 (0.31-2.01) | 0.626 |  |  |
| **PT**, seconds |  |  |  |  |
| > 14.5 vs. ≤ 14.5 | 0.92 (0.48-1.76) | 0.809 |  |  |
| **ALT**, U/L |  |  |  |  |
| > 40 vs. ≤ 40 | 1.90 (1.09-3.32) | **0.024** | 1.73 (0.90-3.31) | 0.099 |
| **AST**, U/L |  |  |  |  |
| > 40 vs. ≤ 40 | 2.19 (1.24-3.86) | **0.007** | 1.58 (0.82-3.05) | 0.176 |
| **ALB**, g/L |  |  |  |  |
| > 35 vs. ≤ 35 | 0.58 (0.26-1.29) | 0.179 |  |  |
| **TBIL**, µmol/L |  |  |  |  |
| > 20 vs. ≤ 20 | 1.14 (0.54-2.43) | 0.733 |  |  |
| **AFP**, ng/mL |  |  |  |  |
| ≥ 400 vs. < 400 | 1.94 (1.09-3.45) | **0.024** | 2.28 (1.27-4.11) | **0.006** |
| **Number of tumors** |  |  |  |  |
| Multiple vs. single | 1.46 (0.78-2.71) | 0.234 |  |  |
| **Tumor diameter**, cm |  |  |  |  |
| > 5 vs. ≤ 5 | 1.69 (0.85-3.38) | 0.135 |  |  |
| **Satellite nodules**, |  |  |  |  |
| Yes vs. no | 1.74 (0.99-3.05) | 0.054 |  |  |
| **Edmondson-Steiner grade**, |  |  |  |  |
| III-IV vs. I-II | 0.80 (0.45-1.43) | 0.449 |  |  |
| **Vascular invasion**, |  |  |  |  |
| Yes vs. no | 0.70 (0.40-1.22) | 0.204 |  |  |
| **Blood loss**, mL |  |  |  |  |
| ≥ 400 vs. < 400 | 0.67 (0.33-1.37) | 0.272 |  |  |
| **Transfusion**, |  |  |  |  |
| Yes vs. no | 0.84 (0.2-3.48) | 0.814 |  |  |
| **Margin**, |  |  |  |  |
| Wide vs. narrow | 0.69 (0.38-1.26) | 0.227 |  |  |
| **Extent of resection**, |  |  |  |  |
| Major vs. minor | 1.28 (0.73-2.25) | 0.396 |  |  |
| **Number of HRRFs** | 1.41 (1.00-2.00) | 0.05 |  |  |
| **PAT**, |  |  |  |  |
| Yes vs. no | 0.39 (0.22-0.70) | **0.002** | 0.33 (0.18-0.60) | **< 0.001** |

Bold values indicate statistical significance (*P* < 0.05).

RFS, recurrence-free survival; HCC, hepatocellular carcinoma; HRRFs, high-risk recurrent factors; PSM, propensity score matching; HR, hazard ratio; CI, confidence interval; HBsAg, hepatitis B surface antigen; HBV-DNA, hepatitis B virus-deoxyribonucleic acid; PLT, platelet; PT, prothrombin time; ALT, alanine aminotransferase; AST, aspartate aminotransaminase; ALB, serum albumin; TBIL, total serum bilirubin; AFP, alpha-fetoprotein; PAT, postoperative adjuvant therapy.
