# Supplementary material for: Postoperative adjuvant tyrosine kinase inhibitors combined with anti-PD-1 antibodies improves surgical outcomes for hepatocellular carcinoma with high-risk recurrent factors
Source: Front Immunol. 2023 Jun 8;14:1202039. doi: 10.3389/fimmu.2023.1202039 (PMC10285103; doi:10.3389/fimmu.2023.1202039)
Supplement: Supplementary file 1 [file DataSheet_1.zip › Supplementary Table 3.DOCX]

**TABLE S3** Univariable and multivariable Cox regression analyses for risk factors associated with OS in HCC patients with HRRFs after PSM.

| **Variable** | **Univariable Analysis** | | **Multivariable Analysis** | |
| --- | --- | --- | --- | --- |
|  | **HR (95% CI)** | ***P*** | **HR (95% CI)** | ***P*** |
| **Gender**, |  |  |  |  |
| Male vs. female | 0.63 (0.22-1.82) | 0.392 |  |  |
| **Age**, years |  |  |  |  |
| ≥ 60 vs. < 60 | 0.87 (0.35-2.16) | 0.763 |  |  |
| **HBsAg**, IU/mL |  |  |  |  |
| ≥ 250 vs. < 250 | 1.12 (0.50-2.49) | 0.783 |  |  |
| **HBV-DNA**, copies/mL |  |  |  |  |
| ≥ 2000 vs. < 2000 | 0.99 (0.42-2.33) | 0.974 |  |  |
| **PLT**, x 10^9^/L |  |  |  |  |
| > 100 vs. ≤ 100 | 1.32 (0.31-5.57) | 0.707 |  |  |
| **PT**, seconds |  |  |  |  |
| > 14.5 vs. ≤ 14.5 | 0.62 (0.24-1.65) | 0.342 |  |  |
| **ALT**, U/L |  |  |  |  |
| > 40 vs. ≤ 40 | 1.43 (0.66-3.13) | 0.366 |  |  |
| **AST**, U/L |  |  |  |  |
| > 40 vs. ≤ 40 | 2.92 (1.37-6.25) | **0.006** | 1.68 (0.70-4.01) | 0.245 |
| **ALB**, g/L |  |  |  |  |
| > 35 vs. ≤ 35 | 0.87 (0.26-2.91) | 0.823 |  |  |
| **TBIL**, µmol/L |  |  |  |  |
| > 20 vs. ≤ 20 | 1.72 (0.69-4.27) | 0.241 |  |  |
| **AFP**, ng/mL |  |  |  |  |
| ≥ 400 vs. < 400 | 2.51 (1.06-5.94) | **0.037** | 2.91 (1.19-7.11) | **0.019** |
| **Number of tumors** |  |  |  |  |
| Multiple vs. single | 1.55 (0.67-3.55) | 0.305 |  |  |
| **Tumor diameter**, cm |  |  |  |  |
| > 5 vs. ≤ 5 | 1.88 (0.71-4.96) | 0.204 |  |  |
| **Satellite nodules**, |  |  |  |  |
| Yes vs. no | 3.73 (1.56-8.91) | **0.003** | 2.76 (1.05-7.22) | **0.039** |
| **Edmondson-Steiner grade**, |  |  |  |  |
| III-IV vs. I-II | 1.40 (0.59-3.32) | 0.444 |  |  |
| **Vascular invasion**, |  |  |  |  |
| Yes vs. no | 0.59 (0.27-1.26) | 0.170 |  |  |
| **Blood loss**, mL |  |  |  |  |
| ≥ 400 vs. < 400 | 1.58 (0.69-3.61) | 0.282 |  |  |
| **Transfusion**, |  |  |  |  |
| Yes vs. no | 3.77 (1.11-12.84) | **0.034** | 3.61 (0.97-13.43) | 0.056 |
| **Margin**, |  |  |  |  |
| Wide vs. narrow | 0.73 (0.32-1.68) | 0.465 |  |  |
| **Extent of resection**, |  |  |  |  |
| Major vs. minor | 1.57 (0.72-3.42) | 0.258 |  |  |
| **Number of HRRFs** | 1.86 (1.14-3.04) | **0.013** | 1.23 (0.72-2.10) | 0.443 |
| **PAT**, |  |  |  |  |
| Yes vs. no | 0.20 (0.07-0.58) | **0.003** | 0.18 (0.06-0.54) | **0.002** |

Bold values indicate statistical significance (*P* < 0.05).

RFS, recurrence-free survival; HCC, hepatocellular carcinoma; HRRFs, high-risk recurrent factors; PSM, propensity score matching; HR, hazard ratio; CI, confidence interval; HBsAg, hepatitis B surface antigen; HBV-DNA, hepatitis B virus-deoxyribonucleic acid; PLT, platelet; PT, prothrombin time; ALT, alanine aminotransferase; AST, aspartate aminotransaminase; ALB, serum albumin; TBIL, total serum bilirubin; AFP, alpha-fetoprotein; PAT, postoperative adjuvant therapy.
