# Supplementary material for: Postoperative adjuvant tyrosine kinase inhibitors combined with anti-PD-1 antibodies improves surgical outcomes for hepatocellular carcinoma with high-risk recurrent factors
Source: Front Immunol. 2023 Jun 8;14:1202039. doi: 10.3389/fimmu.2023.1202039 (PMC10285103; doi:10.3389/fimmu.2023.1202039)
Supplement: Supplementary file 1 [file DataSheet_1.zip › Supplementary Table 4.DOCX]

**TABLE S4** Univariable and multivariable Cox regression analyses for risk factors associated with recurrence of HCC in 250 HCC patients.

| **Variable** | **Univariable Analysis** | | **Multivariable Analysis** | |
| --- | --- | --- | --- | --- |
|  | **HR (95% CI)** | ***P*** | **HR (95% CI)** | ***P*** |
| **Gender**, |  |  |  |  |
| Male vs. female | 1.64 (0.75-3.55) | 0.212 |  |  |
| **Age**, years |  |  |  |  |
| ≥ 60 vs. < 60 | 0.77 (0.47-1.24) | 0.277 |  |  |
| **HBsAg**, IU/mL |  |  |  |  |
| ≥ 250 vs. < 250 | 1.19 (0.77-1.84) | 0.435 |  |  |
| **HBV-DNA**, copies/mL |  |  |  |  |
| ≥ 2000 vs. < 2000 | 1.09 (0.67-1.77) | 0.736 |  |  |
| **PLT**, x 10^9^/L |  |  |  |  |
| > 100 vs. ≤ 100 | 1.48 (0.76-2.87) | 0.245 |  |  |
| **PT**, seconds |  |  |  |  |
| > 14.5 vs. ≤ 14.5 | 1.10 (0.65-1.85) | 0.730 |  |  |
| **ALT**, U/L |  |  |  |  |
| > 40 vs. ≤ 40 | 1.87 (1.20-2.92) | **0.006** | 1.35 (0.77-2.36) | 0.289 |
| **AST**, U/L |  |  |  |  |
| > 40 vs. ≤ 40 | 2.59 (1.66-4.03) | **< 0.001** | 1.56 (0.87-2.77) | 0.133 |
| **ALB**, g/L |  |  |  |  |
| > 35 vs. ≤ 35 | 0.57 (0.28-1.14) | 0.111 |  |  |
| **TBIL**, µmol/L |  |  |  |  |
| > 20 vs. ≤ 20 | 1.36 (0.77-2.42) | 0.294 |  |  |
| **AFP**, ng/mL |  |  |  |  |
| ≥ 400 vs. < 400 | 2.38 (1.55-3.68) | **< 0.001** | 1.39 (0.80 -2.21) | 0.165 |
| **Number of tumors** |  |  |  |  |
| Multiple vs. single | 2.62 (1.63-4.22) | **< 0.001** | 2.09 (1.27-3.43) | **0.004** |
| **Tumor diameter**, cm |  |  |  |  |
| > 5 vs. ≤ 5 | 3.30 (2.02-5.37) | **< 0.001** | 2.06 (1.22-3.48) | **0.007** |
| **Satellite nodules**, |  |  |  |  |
| Yes vs. no | 4.34 (2.79-6.75) | **< 0.001** | 2.92 (1.79-4.77) | **< 0.001** |
| **Edmondson-Steiner grade**, |  |  |  |  |
| III-IV vs. I-II | 1.66 (1.07-2.56) | **0.023** | 0.98 (0.60-1.60) | 0.937 |
| **Vascular invasion**, |  |  |  |  |
| Yes vs. no | 3.32 (2.15-5.13) | **< 0.001** | 1.99 (1.22-3.25) | **0.006** |
| **Blood loss**, mL |  |  |  |  |
| ≥ 400 vs. < 400 | 1.47 (0.84-2.57) | 0.175 |  |  |
| **Transfusion**, |  |  |  |  |
| Yes vs. no | 1.96 (0.85-4.51) | 0.112 |  |  |
| **Margin**, |  |  |  |  |
| Wide vs. narrow | 0.89 (0.56-1.40) | 0.611 |  |  |
| **Extent of resection**, |  |  |  |  |
| Major vs. minor | 2.63 (1.67-4.12) | **< 0.001** | 1.25 (0.77-2.05) | 0.367 |
| **PAT**, |  |  |  |  |
| Yes vs. no | 1.35 (0.78-2.31) | 0.280 |  |  |

Bold values indicate statistical significance (*P* < 0.05).

HCC, hepatocellular carcinoma; HR, hazard ratio; CI, confidence interval; HBsAg, hepatitis B surface antigen; HBV-DNA, hepatitis B virus-deoxyribonucleic acid; PLT, platelet; PT, prothrombin time; ALT, alanine aminotransferase; AST, aspartate aminotransaminase; ALB, serum albumin; TBIL, total serum bilirubin; AFP, alpha-fetoprotein; PAT, postoperative adjuvant therapy.
